# Cross-Neutralization of SARS-CoV-2-Specific Antibodies in Convalescent and Immunized Human Sera against the Bat and Pangolin Coronaviruses

**DOI:** 10.3390/v14081793

**Published:** 2022-08-16

**Authors:** Kanjana Srisutthisamphan, Janya Saenboonrueng, Asawin Wanitchang, Ratchanont Viriyakitkosol, Anan Jongkaewwattana

**Affiliations:** 1 Virology and Cell Technology Laboratory, National Center for Genetic Engineering and Biotechnology (BIOTEC), National Science and Technology Development Agency (NSTDA), Pathumthani 12120, Thailand; 2 Veterinary Health and Innovation Management Research Group, National Center for Genetic Engineering and Biotechnology (BIOTEC), National Science and Technology Development Agency (NSTDA), Pathumthani 12120, Thailand

**Keywords:** pangolin coronavirus, bat coronavirus, SARS-CoV-2, neutralizing antibodies, chimeric proteins, pseudo-virus

## Abstract

Coronaviruses isolated from bats and pangolins are closely related to SARS-CoV-2, the causative agent of COVID-19. These so-called sarbecoviruses are thought to pose an acute pandemic threat. As SARS-CoV-2 infection and vaccination have become more widespread, it is not known whether neutralizing antibodies to SARS-CoV-2 can cross-neutralize coronaviruses transmitted by bats or pangolins. In this study, we analyzed antibody-mediated neutralization with serum samples from COVID-19 patients (*n* = 31) and those immunized with inactivated SARS-CoV-2 vaccines (*n* = 20) against lentivirus-based pseudo-viruses carrying the spike derived from ancestral SARS-CoV-2, bat (RaTG13 or RshSTT182), or pangolin coronaviruses (PCoV-GD). While SARS-CoV-2, PCoV-GD, and RshSTT182 spikes could promote cell-cell fusion in VeroE6 cells, the RaTG13 spike did not. RaTG13, on the other hand, was able to induce cell-cell fusion in cells overexpressing ACE2. Dramatic differences in neutralization activity were observed, with the highest level observed for RaTG13, which was even significantly higher than SARS-CoV-2, PCoV-GD, and RshSTT182 pseudo-viruses. Interestingly, pseudo-viruses containing the chimeric protein in which the receptor-binding domain (RBD) of PCoV-GD spike was replaced by that of RaTG13 could be strongly neutralized, whereas those carrying RaTG13 with the RBD of PCoV-GD were significantly less neutralized. Because the high neutralizing activity against RaTG13 appears to correlate with its low affinity for binding to the human ACE2 receptor, our data presented here might shed light on how pre-existing immunity to SARS-CoV-2 might contribute to protection against related sarbecoviruses with potential spillover to the human host.

## 1. Introduction

As of July 2022, the pandemic caused by the 2019 coronavirus disease (COVID-19) outbreak has caused more than 6 million deaths worldwide. The causative agent of COVID-19, severe acute respiratory syndrome coronavirus type 2 (SARS-CoV-2), belongs to the *sarbecovirus* subgenus of the *beta-coronavirus* genus, which is mainly found in horseshoe bats [1,2]. While the intermediate host of SARS-CoV-2 has not yet been identified, two bat coronaviruses have been proposed as its closest relatives. The first is RaTG13, which was isolated from the horseshoe bat in 2013 [3]. The other virus, RmYN02, was identified by metagenomic sequencing of viruses isolated from *R. malayanus*, the Malayan horseshoe bat [4]. Recently, Malayan pangolins (*Manis javanica*) have been found to harbor coronaviruses closely related to SARS-CoV-2. In fact, structural analyses of the spike and its interaction with ACE2, which revealed the position in the receptor binding domain (RBD) of the residues responsible for the interaction between the two proteins, showed that the RBD of the pangolin-CoV spike shares the highest degree of similarity (up to 98.6%) with that of SARS-CoV-2, even more than those of bat coronaviruses. [5]. Indeed, pangolins have recently been suggested as a potential reservoir for mammalian CoV in some studies [6,7]. It has even been speculated that recombination between bat CoV and others, particularly those found in pangolins, could potentially lead to the emergence of SARS-CoV-2 [8].

Since sarbecoviruses from bats and pangolins can enter human cells via an ACE2-dependent mechanism [9], it is possible that these viruses can cross the species barrier and infect human hosts [10]. Numerous studies have provided serological evidence for the spread of bat CoV to human populations, especially in rural communities with frequent wildlife contact [7,11,12]. In addition, bat CoVs closely related to SARS-CoV-2 have recently been identified in rural areas in Southeast Asia, including Thailand and Cambodia [13,14]. Given the ongoing pandemic of SARS-CoV-2, it is plausible to hypothesize that pre-existing immunity to SARS-CoV-2 could at least partially prevent the spread of other sarbecoviruses. One unanswered question is whether the specific antibodies to SARS-CoV-2 found in the convalescent sera from COVID-19 patients could cross-neutralize coronaviruses from bats and pangolins.

In this study, we sought to determine whether human sera with SARS-CoV-2 neutralizing activity could also cross-neutralize pangolin or bat-derived CoV pseudo-viruses. To our surprise, the RaTG13 spike could be strongly neutralized by all tested sera despite having an RBD that was only distantly related to that of SARS-CoV-2. In contrast to the native SARS-CoV-2 spike, pseudo-viruses containing spikes of other sarbecoviruses were less strongly neutralized. Moreover, using pseudo-viruses with chimeric spike proteins, we demonstrated that the strong neutralizing activity was caused by the RBD of RaTG13. Overall, our study sheds light on the mechanism by which neutralizing antibodies against the SARS-CoV-2 spike may also cross-neutralize other spikes that are closely related. This information may be useful for the future development of vaccines to prevent the spread of zoonoses, especially those caused by sarbecoviruses similar to SARS-CoV-2.

## 2. Materials and Methods

### 2.1. Cells

Human embryonic kidney cells HEK 293T/17 (ATCC-CRL-1573), African green monkey kidney cells (VeroE6, ATCC-CRL-1586), and MA-104 (ATCC- CRL-2378.1) were cultured in Opti-MEM (Thermo Scientific, Waltham, MA, USA) at 37 °C and 5% CO_2_. All culture media were supplemented with 10% fetal bovine serum and antibiotic/mycotic (Thermo Scientific). HEK293T/17 cells stably expressing the codon-optimized human angiotensin-converting enzyme-2 (ACE-2) (HEK293T-ACE2) were prepared by retroviral transduction as previously described [15]. A549-ACE2 cells were obtained commercially (InvivoGen, San Diego, CA, USA) and cultured and maintained according to the manufacturer’s instructions.

### 2.2. Serum Samples

Sera (*n* = 31) from COVID-19 patients who became ill during the outbreak of the original SARS-CoV-2 strain (Wuhan-like) in Thailand from April to October 2020 were generously provided by Thailand’s National Institute of Health (NIH) and Siriraj Hospital. Taksin Hospital, Bangkok Thailand, provided vaccinated sera (*n* = 20) from healthy individuals one month after vaccination twice with inactivated vaccines (CoronaVac). The NIH also provided pre-pandemic sera (*n* = 10). All serum samples were stored at −20°C until they were used.

### 2.3. Plasmid Constructs

The full-length spike of SARS-CoV-2 (Wuhan-Hu-1; Genbank: MN908947), bat CoVs, including RaTG13 (Genbank: MN996532.2) and RshSTT182 (GISAID: EPI_ISL_852604), and pangolin-CoV (PCoV-GD; GISAID: EPI_ISL_410721) were codon-optimized for high expression in mammalian cells, synthesized, and cloned into the cloning vector pUC57 (Genscript, Piscataway NJ, USA). The synthetic gene was designed to be flanked by MluI and NotI restriction sites to facilitate cloning into the pCAGGS expression plasmid, which was modified to contain both restriction sites. The chimeric spikes were produced by a ligation-independent strategy using the In-Fusion cloning kit (Takara Bio, Mountain View, CA, USA). The expression plasmid harboring human TMPRSS2 was also constructed from a synthetic gene using the same strategy. All plasmids were verified by direct nucleotide sequencing to ensure that no unwanted mutations occurred during cloning.

### 2.4. Pseudo-Virus Preparation and Titration

With minor modifications, lentiviral pseudo-viruses carrying CoV spike were produced as previously described by Di Genova et al. [16]. Briefly, to generate pseudo-viruses, a combination of plasmids was used, including the lentivirus backbone expressing a firefly luciferase reporter gene (pCSFLW), the expression plasmid expressing HIV-1 structural/regulatory proteins (pCMVR8.91), and pCAGGS expressing each spike construct. Unless otherwise specified, HEK293T/17 production cells were seeded in 6-well plates 24 h before transfection with the following plasmids at 7.5 × 10^5^ cells/well: 600 ng pCMVR8.91, 600 ng pCSFLW, and 500 ng pCAGGS spike in OptiMEM containing 10 µL poly-ethylenimine (PEI). Transfected cells were incubated at 37 °C with 5% CO_2_. Cells were washed and cultured in DMEM containing 10% FBS at 12 h after transfection. At 72 h after transfection, pooled supernatants containing pseudo-viruses were collected, centrifuged at 1500× *g* for 10 min at 4 °C to remove cell debris, aliquoted, and stored at −80 °C.

To titrate pseudo-viruses, HEK 293T/17-ACE2 cells were first transfected with the expression plasmid encoding human TMPRSS2 using Fugene HD (Promega, Madison, WI, USA) according to the manufacturer’s instructions. At 24 h after transfection, the supernatants were replaced by DMEM containing 10% FBS and then used as pseudo-virus target cells. The supernatants containing pseudo-viruses were serially diluted two-fold in DMEM medium in 96-well culture plates, and TMPRSS2-expressing HEK 293T/17-ACE2 target cells (1 × 10^4^ cells/well) were added to each well. After 72 h, the luminescence of the cell cultures (in Relative Luminescence Units or RLUs) was evaluated by luminometry (Agilent, Santa Clara, CA, USA) using the Bright-Glo Assay System (Promega, Madison, WI, USA).

### 2.5. Cell-Cell Fusion Assay

VeroE6 or HEK293T-ACE2 cells (5 × 10^5^ cells/mL) were seeded in a 6-well plate in Opti- MEM, supplemented with 10% FBS. After 24 h, cells were washed with 1× PBS and maintained in media without FBS. Cells were then transfected with pCAGGS expressing spike (1.5 µg) using Fugene HD (Promega) according to the manufacturer’s protocol. The media containing the transfection mixture were removed 8 h after transfection (hpt). Cells were then maintained in media containing recombinant trypsin (2 µg/mL) (Thermo Scientific). At 24 h after transfection, cells were examined for cell-cell fusion under an inverted light microscope.

### 2.6. Pseudo-Virus-Based Neutralization Assay

To determine the neutralizing activity of the serum samples, a two-fold serial dilution of heat-inactivated sera was performed starting at 1:40, in a culture medium (high-glucose DMEM without FBS). Sera were mixed with pseudo-viruses containing the CoV spike of interest at a ratio of 1:1 vol/vol in a 96-well culture plate. The pseudo-virus input used was normalized to 1 × 10^5^ RLU/well. The serum-pseudo-virus mixture was then incubated at 37 °C for 1 h. Cell suspensions of HEK293T-ACE-2 pre-transfected with pCAGGS expressing human TMPRSS2 (2 × 10^4^ cells/mL) were then mixed with the serum-pseudo-virus mixture and seeded into each well of CulturPlate™ Microplates (PerkinElmer, Waltham, MA, USA). Plates were incubated at 37 °C for 48 h, and neutralizing antibodies were determined by luciferase activity, as previously described [17].

### 2.7. Western Blot Analysis

HEK293T/17 cells were transfected with Fugene HD (Promega) with plasmids expressing different spike variants. At 48 h after transfection, cells were lysed with the mammalian cell lysis buffer (50 mM Tris HCl pH 8.0, 100 mM NaCl, 2 mM DTT, 5 mM EDTA, 0.5% NP −40, and protease inhibitors). Total cell lysates were separated using SDS-PAGE and proteins were then transferred to nitrocellulose membranes (Biorad Laboratories, Hercules, CA, USA). The membrane was blocked with 5% dry milk powder and probed with rabbit polyclonal anti-SARS-CoV-2 spike antibodies (Sino Biological, Beijing, China) or mouse monoclonal anti-β-actin antibodies (Cell Signaling). Membranes were then incubated with horseradish peroxidase (HRP)-conjugated goat anti-mouse IgG (1:5000; Abcam, Cambridge, UK). Target proteins were visualized using Clarity Western ECL substrate (Biorad Laboratories). For ACE2 detection, mouse monoclonal anti-human ACE2 antibodies (Thermo Scientific) were used as primary antibodies.

### 2.8. Statistical Analysis

GraphPad Prism 9.0 (GraphPad Software, San Diego, CA, USA) was used for statistical analyses.

## 3. Results

### 3.1. SARS-CoV-2, Bat, and Pangolin Expression CoV Spikes and the Cell–Cell Fusion Characteristics Induced by These Spikes in Transfected Cells

**Figure 1:**
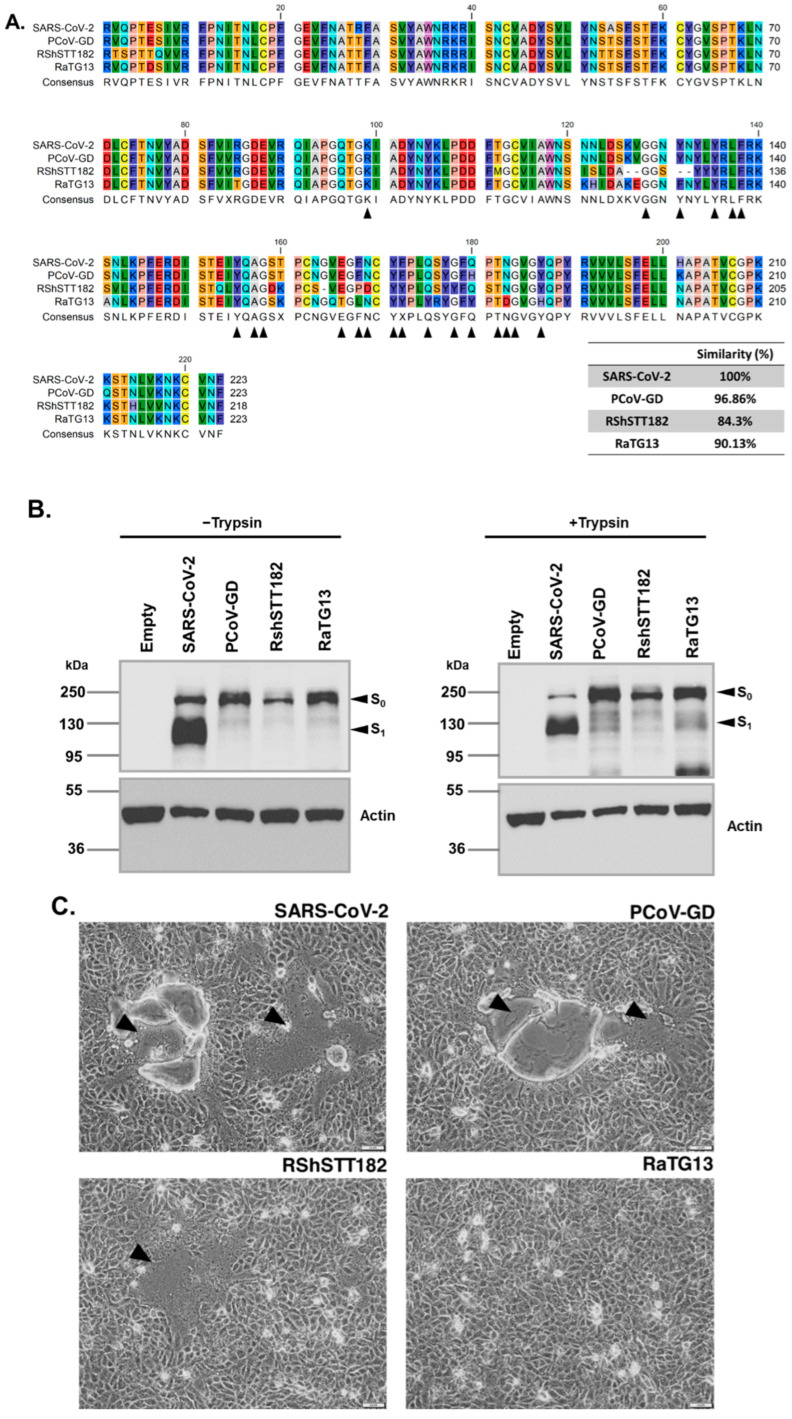
Expression and cell-cell fusion activity of sarbecovirus spikes in VeroE6 cells. (**A**). The sequence alignment of the RBD of SARS-CoV-2 and that of PCoV-GD, RShSTT182, and RaTG13. Black triangles indicate interacting amino acids of the RBD of SARS-CoV-2 with hACE2. Alignment was performed using QIAGEN CLC Genomics Workbench and percent similarity was analyzed using the web-based platform SIAS (Universidad Complutense de Madrid). (**B**). Expression of each spike variant in HEK-293T cells in the absence and presence of trypsin. The unprocessed spike (S0) and the cleaved spike product (S1) are indicated by arrows. Expression of beta-actin served as a loading control. (**C**). Cell–cell fusion in VeroE6 cells transfected with each spike variant in the presence of trypsin. Images were taken under an inverted light microscope 24 h after transfection (100-μm scale bars). Black arrows mark representative areas where cell–cell fusion was observed.

The spike constructs used in this study include human CoV (SARS-CoV-2), bat CoV (RaTG13 and RshSTT182), and pangolin CoV (PCoV-GD). As shown in Figure 1A, sequence alignment of the receptor binding domain (RBD) revealed that the RBD of PCoV-GD had the highest similarity (96.86%) to that of SARS-CoV-2. However, the RBD of RaTG13 and RshSTT182 showed quite distant similarity (90.13% and 84.3%, respectively). All constructs in this study were codon-optimized for high expression in human cells and were strongly expressed in transfected cells, as shown in Figure 1B. Remarkably, only the spike of SARS-CoV-2 showed the cleaved product of the S1 subunit, most likely due to the presence of the polybasic furin cleavage site in the construct. However, when the transfected cells were treated with trypsin, the presence of S1 was observed in all constructs (Figure 1B). Interestingly, when each spike construct was expressed in VeroE6 cells in the presence of trypsin, massive syncytium formation occurred in cells expressing the spike of SARS-CoV-2, RshSTT182, and PCoV-GD but not RaTG13 (Figure 1C). 

### 3.2. The RaTG13 RBD Is Responsible for Its Inability to Induce Cell–Cell Fusion in VeroE6 Cells

**Figure 2:**
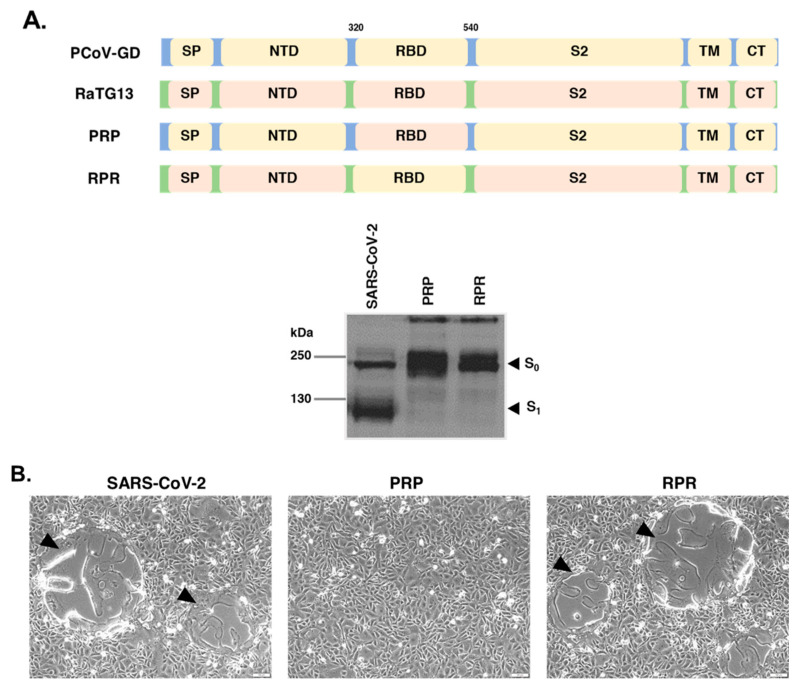
The inability of RaTG13 to undergo cell–cell fusion with VeroE6 cells is due to its RBD. (**A**). Schematic illustration of chimeric spike constructs in which the RBD of RaTG13 was swapped with that of PCoV-GD (PRP) and the RBD of PCoV-GD was swapped with that of RaTG13 (RPR). Using antibodies specific for the SARS-CoV-2 spike, the expression of the two chimeric constructs was evaluated using Western blot. Arrows indicate the unprocessed spike (S0) and the cleaved spike product (S1). (**B**). Cell–cell fusion in the presence of trypsin in VeroE6 cells transfected with each chimeric spike construct. At 24 h after transfection, images were captured using an inverted light microscope (scale bars of 100 µm). The black arrows indicate areas where cell–cell fusion was observed.

It is noteworthy that while RshSTT182 appears to be less conserved with respect to the overall RBD structure, specific residues known to play a crucial role in binding human ACE2 are more conserved in RshSTT182 than in RaTG13 (Figure 1A; black triangles). To determine whether the RBD of the RaTG13 spike is responsible for the inability to undergo cell–cell fusion in VeroE6 cells, we generated chimeric constructs in which the RBD of RaTG13 was replaced by that of PCoV-GD (designated RPR) and the RBD of PCoV-GD was replaced by that of RaTG13 (designated PRP). Figure 2A depicts the schematic representation of each chimeric spike and its expression in transfected HEK293T cells. As expected, no syncytium was observed when VeroE6 cells were transfected with the plasmid expressing the PRP chimeric spike construct, whereas cells transfected with the RPR chimeric spike construct showed robust cell–cell fusion (Figure 2B). These results suggest that the RBD of RaTG13 is responsible for the inability of the transfected VeroE6 cells to form syncytia.

### 3.3. RaTG13 Spike Could Induce Cell–Cell Fusion in Human ACE2-Overexpressing Cells

**Figure 3:**
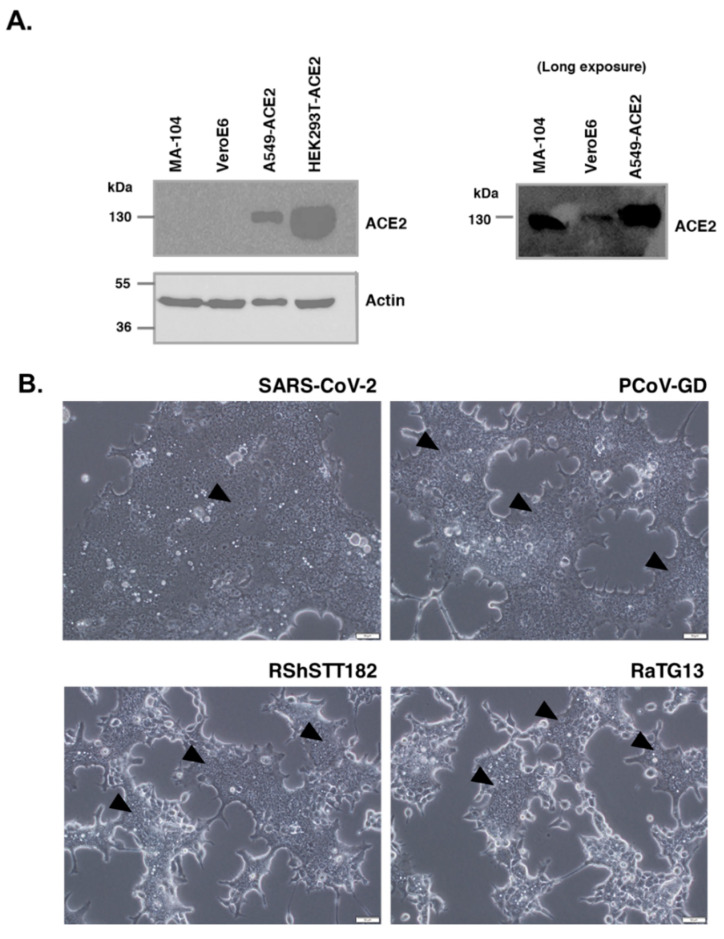
Spike of RaTG13 could trigger cell–cell fusion in cells strongly expressing human ACE2. (**A**). The level of ACE2 expression in cell lines susceptible to SARS-CoV-2 infection. Using antibodies against human ACE2, Western blot analysis of lysates from each cell line was performed. Beta-actin expression served as a loading control. To detect ACE2 expression in VeroE6 cells, a longer exposure time was used and the lane of HEK-293T-ACE2 was excluded. (**B**)**.** Cell–cell fusion in HEK293T-ACE2 cells transfected with each variant of the spike protein in the presence of trypsin. Images were taken 24 h following transfection using an inverted light microscope (scale bar of 100 µm). The black arrows indicate areas where cell–cell fusion was observed.

To determine whether the failure of the RaTG13 spike to induce cell–cell fusion in VeroE6 cells was due to the low expression of ACE2 in this cell line, we examined the properties of the RaTG13 spike in HEK-293T cells modified to overexpress human ACE2 (HEK293T-ACE2). After Western blot analysis, HEK-293T-ACE2 showed significantly higher ACE2 expression than other cell lines known to be susceptible to SARS-CoV-2 infection, including VeroE6, MA-104, and A549-ACE2 (Figure 3A). Of note, VeroE6 cells had the lowest expression of ACE2 among all cells examined in this study (Figure 3A). After HEK293T-ACE2 cells were transfected with a plasmid expressing RaTG13 spike and cultured with trypsin, cell–cell fusion was observed in the transfected cells (Figure 3B). These results indicate that the RaTG13 spike can be cleaved by trypsin and interact with higher expression of human ACE2, resulting in cell–cell fusion. In addition, it should be noted that the extent of cell fusion activity of RaTG13 in HEK-293T-ACE2 cells was significantly lower than that induced by the spike of SARS-CoV-2 and PCoV-GD (Figure 3B).

### 3.4. Pseudo-Viruses with Different Sarbecovirus Spikes Were Able to Enter HEK293T-ACE2 Cells with Variable Efficiency

**Figure 4:**
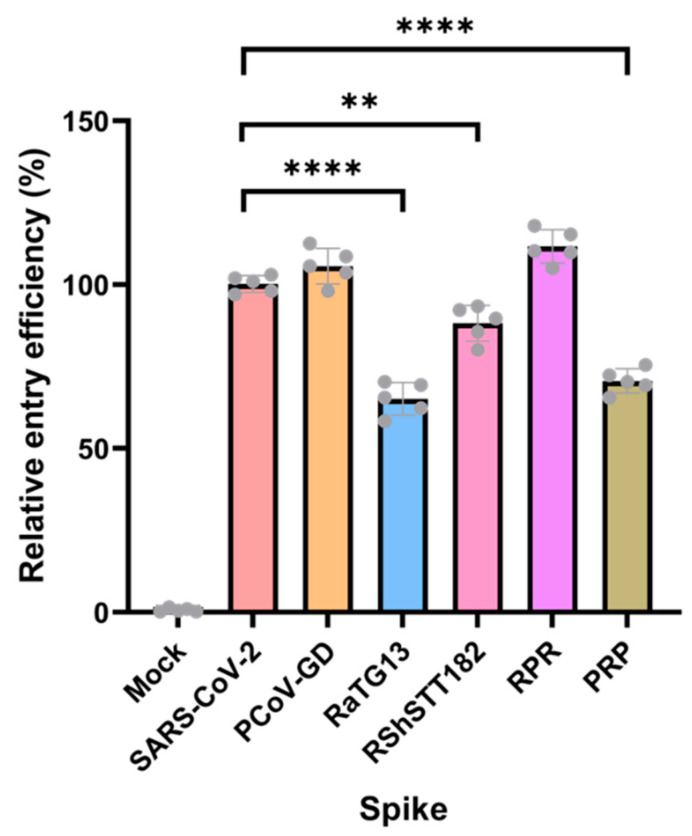
Entry of pseudo-viruses carrying each spike into HEK-293T-ACE2 cells. After pseudo-virus entry, the luciferase signal was used to evaluate the entry efficiency. The entry efficiency of SARS-CoV-2 wild-type pseudo-viruses was taken as 100%. The error bars depict the standard deviation (*n* = 5) with each dot representing each replication. Normalization of the relative luminescence unit (RLU) of Luc reporter gene expression to the p24 content of the pseudo-viruses. Adjusted *p* values were calculated using one-way ANOVA: ** *p* 0.01, **** *p* 0.0001.

The finding that the spike of RaTG13 could not trigger cell–cell fusion in VeroE6 but could do so in HEK293T-ACE2 cells led us to speculate that the RaTG13 spike may not enter host cells as efficiently as other CoVs. To test this hypothesis, we generated lentivirus-based pseudo-viruses bearing each spike construct and assessed their entry efficiency into HEK293T/17-ACE2 in the presence of TMPRSS2. As shown in Figure 4, the infectivity of the pseudo-viruses bearing spike of RaTG13 and the PRP chimera were substantially lower than those of SARS-CoV-2, PCoV-GD, RshSTT182 and the RPR chimera. Interestingly, the infectivity of that bearing PCoV-GD and RPR were slightly higher than that of SARS-CoV-2. Remarkably, this pattern was independent of the pseudo-viruses’ input dose. These findings suggest that the RBD of RaTG13 and the PRP could still bind to the human ACE2 receptor, albeit weakly compared with other spikes, resulting in lower infectivity of the pseudo-typed virus. It is also worth noting that we attempted to investigate the entry of the pseudo-viruses into VeroE6 cells but were unable to obtain sufficient luciferase activity for comparison. Because VeroE6 cells were not readily transfected by pCAGGS-TMPRSS2, insufficient TMPRSS2 could result in low transduction efficiency of the pseudo-viruses.

### 3.5. SARS-CoV-2 Positive Sera Exhibit Varying Levels of Neutralizing Activity against Bat and Pangolin CoVs

**Figure 5:**
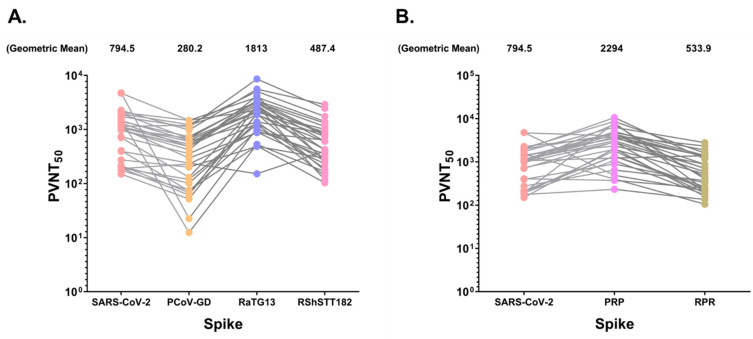

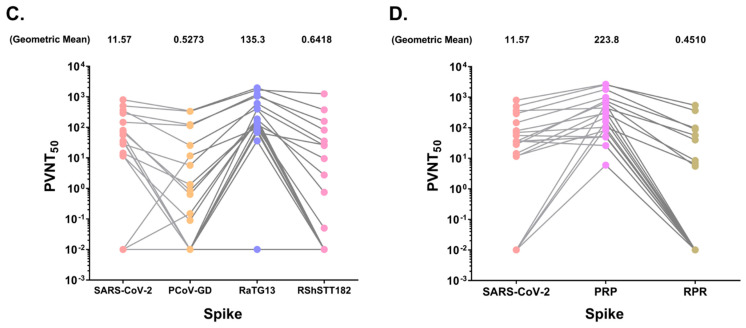
Analyses of the cross-neutralization activity of SARS-CoV-2-positive serum samples against pseudo-viruses carrying each sarbecovirus spike. (**A**). Neutralization titers against SARS-CoV-2, PCoV-GD, RaTG13, and RshSTT182 in 31 convalescent donors. (**B**). Neutralization titers of convalescent sera against pseudo-viruses carrying a chimeric PRP and RPR spike. (**C**). Neutralization titers of 20 donors vaccinated with CoronaVac against SARS-CoV-2, PCoV-GD, RaTG13, and RshSTT182 one month after vaccination. (**D**). Titers of neutralization of vaccinated sera against pseudo-viruses with chimeric PRP and RPR spike. The geometric mean of each data set is shown in all analyses.

We next investigated the possibility of cross-neutralizing effects against pseudo-viruses carrying spikes of the individual sarbecoviruses in SARS-CoV-2-positive sera from convalescent COVID-19 patients or those previously vaccinated with inactivated vaccines. Importantly, we selected and tested sera from COVID-19 patients who had recovered from the initial pandemic wave in order to compare their reactivity with that of individuals who had received the ancestral strain-based CoronaVac vaccine. Because the RBD of PCoV-GD is more similar to that of SARS-CoV-2 than to that of RaTG13, we initially expected that some sera would neutralize the RaTG13 pseudo-virus less effectively than others. Surprisingly, we found that convalescent sera neutralized RaTG13 pseudo-virus approximately 2.3-fold more effectively than SARS-CoV-2 pseudo-virus (Figure 5A). Compared with the SARS-CoV-2 pseudo-virus, the neutralizing activity of the PCoV-GD and RshSTT182 pseudo-viruses decreased 2.83- and 1.58-fold, respectively (Figure 5A). When the sera were tested against the pseudo-virus carrying the PRP chimeric spike, a 2.88-fold increase in neutralizing activity was observed (Figure 5B). As expected, the sera neutralized the RPR chimeric spike pseudo-virus more effectively than PCoV-GD but less so than RaTG13. These findings suggest that the RBD, as opposed to other regions of RaTG13’s spike, is responsible for the high neutralizing activity observed in SARS-CoV-2-positive sera. Notably, we also tested all pseudo-viruses with pre-pandemic sera and none of the tested sera exhibited neutralizing activity (data not shown).

Consistent with the trend observed with convalescent sera, sera from individuals vaccinated with the inactivated SARS-CoV-2 vaccine one month after the second dose appeared to neutralize RaTG13 pseudo-virus more effectively than sera from individuals vaccinated with wild-type SARS-CoV-2, RshSTT182, or PCoV-GD, respectively (Figure 5C). Of note, the neutralizing activity observed in this panel of sera was significantly lower than that observed in the convalescent sera, which demonstrated a remarkable increase in the neutralizing activity of pseudo-virus RaTG13 compared with those spiked with SARS-CoV-2 (11.69-fold), RshSTT182 (210.8-fold), and PCV-GD (13.0-fold) (256.6-fold). When tested with the pseudo-viruses PRP and RPR, the difference in neutralizing activity between PRP and RPR could be as high as 496-fold (Figure 5D). In contrast to other bat or pangolin sarbecoviruses, the RaTG13 pseudo-virus was strongly neutralized by the sera of those immunized with SARS-CoV-2 inactivated vaccines.

## 4. Discussion

Since the emergence of SARS-CoV-2 and its efficient human-to-human transmission, numerous related beta-coronaviruses, particularly those of the *sarbecovirus* subgenus, have been the focus of intense research due to concerns that these pathogens may cross the species barrier and infect human hosts in the same manner as SARS-CoV-2. Because SARS-CoV-2 uses ACE2 as its primary receptor for cell entry, other viruses that use ACE2 for host cell entry are also thought to pose a risk for transmission to humans. While it is well known how the SARS-CoV-2 spike interacts with ACE2 via its RBD, it is not clear whether this knowledge can be applied to other beta-coronaviruses. This study examines the effect of SARS-CoV-2-exposed human sera on the interaction between human ACE2 and the spike of several sarbecoviruses, including bat (RaTG13, RshSTT182) and pangolin (PCoV-GD) coronaviruses.

In contrast to SARS-CoV-2, which contains a polybasic cleavage site at the S1/S2 junction [18,19,20], bat and pangolin coronaviruses possess only a single basic residue that can be cleaved by a trypsin-like enzyme [21,22]. The extensive cell fusion observed in VeroE6 cells expressing SARS-CoV-2 spike in the absence of exogenous trypsin suggests that SARS-CoV-2 spike can efficiently bind to human ACE2 and trigger rapid cell fusion. Since TMPRSS2 has been shown to be absent in VeroE6 cells [23], it is not known which protease(s) cleaved S2’ in our experimental setup. Other proteases, including matrix metalloproteases such as ADAM10 and ADAM17 [24], have been proposed as potential SARS-CoV-2 spike-cleaving enzymes that facilitate not only virus entry but also syncytium formation independent of TMPRSS2. In contrast to the SARS-CoV-2 spike, both bat and pangolin spikes failed to induce cell–cell fusion in cells transfected with the spike plasmid in the absence of exogenous trypsin. This suggests that the S1/S2 junction of these spike proteins may be cleaved by trypsin in a manner similar to the furin-mediated cleavage of the SARS-CoV-2 spike. Since spikes from bat and pangolin coronavirus spikes were able to induce cell fusion in VeroE6 and HEK-293T ACE2 cells, it is reasonable to assume that all spikes are capable of binding to human ACE2 and initiating cell fusion. Notably, all spikes, when pseudo-typed with lentivirus, were able to induce entry of the pseudo-virus into HEK-293T-ACE2 cells, supporting the data that all spikes examined in this study were able to enter cells in an ACE2-dependent manner.

In this study, we demonstrated that SARS-CoV-2-positive sera neutralize bat and pangolin spikes to varying degrees and that this does not directly correlate with the degree of similarity between the RBDs of the respective viruses and SARS-CoV-2. While the RBDs of PCoV-GD and SARS-CoV-2 are very similar, those of RaTG13 and RshSTT182 are more distantly related. It is expected that the pseudo-virus carrying RaTG13 and RshSTT182 will be less neutralized than PCoV-GD, while SARS-CoV-2 will be the most neutralized. To our surprise, most, although not all, sera were able to effectively neutralize the RaTG13 spike-bearing pseudo-virus, and their neutralizing activity was significantly greater than those observed with pseudo-virus bearing the SARS-CoV-2 spike. Our results are consistent with a recent finding by Cantoni et al. [25] that SARS-CoV-2-specific immune sera effectively neutralize RaTG13 pseudo-types. In addition, Liu et al. have shown that SARS-CoV-2 antibodies can cross-react with the RaTG13 RBD, although the binding activity is slightly lower for RaTG13 than for SARS-CoV-2. However, despite the lower binding activity, all sera were able to strongly neutralize pseudo-viruses carrying the RaTG13 spike, whereas only 30% of sera were able to neutralize those carrying the SARS-CoV-2 spike [26]. Of note, the neutralizing activity of SARS-CoV-2-positive sera against RshSTT182 pseudo-viruses was significantly lower than against RaTG13 pseudo-viruses, despite the fact that both are bat coronaviruses.

Our results suggest that the RBD of RaTG13 is responsible for the strong neutralizing activity of SARS-CoV-2-positive sera. In addition, we found that sera from individuals who had received the CoronaVac inactivated virus vaccine neutralized RaTG13 pseudo-viruses in a comparable and potent manner. Since pre-pandemic sera lacked neutralizing activity against RaTG13 pseudo-viruses, it is improbable that pre-existing antibodies to RaTG13 spike are responsible for the observed high neutralizing activity. Notably, the results of this study indicate that the RBD of RaTG13 can be effectively neutralized by SARS-CoV-2 specific sera despite differences in amino acid composition. This may be possibly due to a weaker interaction between the RaTG13 spike and the human ACE2 receptor compared to the other spikes examined in this study. In fact, it has been demonstrated that the binding affinity between the RaTG13 RBD and hACE2 is up to 70-fold lower than that between the SARS-CoV-2 RBD and hACE2 [26]. Consequently, the number of antibodies required to block the interaction between the SARS-CoV-2 spike and the ACE2 receptor may be greater than the number of antibodies required to block the interaction between the RaTG13 spike and the hACE2 receptor. Of note, although the overall similarity between the RshSTT182 RBD and the SARS-CoV-2 RBD is lower than that of RaTG13, key residues responsible for the ACE interaction appear to be more conserved in RshSTT182, possibly resulting in a higher affinity of the RshSTT182 RBD for hACE2 than that of RaTG13. To support this hypothesis, it has been observed that the RaTG13 spike with the mutation T403R, which has been shown to increase human ACE2 binding affinity [27], was less neutralized by the SARS-CoV-2 sera than the native RaTG13 spike [25]. However, it is noteworthy to remark that, in addition to the lesser affinity to ACE2, other factors, particularly the antigenic epitopes that are retained between SARS-CoV-2 and RaTG13 spike, may also contribute to the higher neutralization reported for SARS-CoV-2 sera against RaTG13.

Our approach to analyzing the neutralizing activity against RaTG13 by SARS-CoV-2 positive sera is limited since we only examined the neutralizing activity in individuals exposed to the original strain of SARS-CoV-2. Given that many people are infected with Omicron variants such as BA.1, BA.2, or BA.4/BA.5, which are known to be poorly neutralized by convalescent sera of pre-Omicron patients, it is likely that the neutralizing activity of those exposed to these variants will differ from our findings in this study. To test the neutralizing activity of Omicron-specific sera, however, sera from unvaccinated subjects who have recently recovered from Omicron infection are required, and these are very rare. Further animal experiments should instead be conducted to evaluate whether RaTG13 can be neutralized by Omicron-specific sera as opposed to other variants. Another limitation of this study is that we did not assess the neutralizing activity of the sera over time, as the persistence of antibodies could have a direct effect on cross-neutralization to different sarbecoviruses. These points should also be addressed in future studies.

In conclusion, this study demonstrated that pre-existing immunity from the SARS-CoV-2 vaccine or natural infection could neutralize similar sabecoviruses with a spike that employs human ACE2 as the receptor. This information highlights the significance of immunization against SARS-CoV-2 for preventing not only the current outbreaks but also the likely future spread of additional coronaviruses.

## Data Availability

The data presented in this study are available on request from the corresponding author.
